# Toward a Living Meta-Framework for Evaluating Generative AI in Mental Health

**DOI:** 10.2196/89211

**Published:** 2026-10-15

**Authors:** Ashleigh Sarah Golden, Elias Aboujaoude

**Affiliations:** 1 Department of Psychiatry and Behavioral Sciences, School of Medicine, Stanford University, 401 Quarry Road, Stanford, CA, CA94305, United States, 1 (650) 498-9111; 2 Department of Biomedical Sciences, Cedars-Sinai Medical Center, 8700 Beverly Blvd, Los Angeles, CA, 90048, United States, 1 (310) 423-3277

**Keywords:** generative AI, AI, mental health, ethics, framework, evaluation, framework for AI tool assessment, FAITA, regulation

## Abstract

The proliferation of evaluative approaches for generative AI tools used for mental health purposes reflects much-needed attention to safety, ethics, and emerging risks. However, limited alignment across frameworks complicates comparisons, slows progress toward shared goals, and provides little clarity for regulatory processes. The result is a fragmented field that lacks cohesive guidance as the technology and its real-world patterns of use continue to evolve. This position paper synthesizes current evaluative approaches, outlines sources of divergence across existing frameworks, and identifies shared domains that could serve as anchors for coordinated development. We advocate for a living, flexible meta-framework maintained by a global, cross-sector consortium that can harmonize criteria, support consistent evaluation across jurisdictions, and adapt alongside scientific, clinical, and technological advances. Such a structure can strengthen safety, transparency, and methodological consistency while giving researchers, clinicians, developers, and policymakers a shared foundation for assessing generative AI tools in mental health care.

## The Shifting Landscape of Digital Mental Health Tools

### Background

The digital mental health landscape has expanded rapidly with the advent of large-scale generative AI (GenAI) tools [1]. A 2024 analysis of OpenAI’s GPT Store, which allows users to publish their own chatbots, found that many were promoted as offering emotional support [2]. Mental health practitioners are already navigating the ethical and practical implications of these tools as they quickly reshape how care is delivered, supervised, and taught [3]. Meanwhile, many individuals continue to rely on general-purpose large language models (LLMs), such as ChatGPT (OpenAI), for psychological support. In a 2025 cross-sectional survey of 499 US adults with self-identified mental health conditions recruited through a nonprobability online sample, 48.7% reported using tools like ChatGPT for mental health help [4]. Estimates of such use vary widely with sampling method and how mental health support is defined. A narrative review of available surveys and industry reports estimated that roughly 27% of users have turned to AI for mental health support, while cautioning that inconsistent definitions and unverified online respondents preclude exact figures [5]. Population-representative data are beginning to emerge: in a nationally representative survey conducted in late 2025, 19.2% of US adolescents and young adults reported having used AI chatbots for mental health advice, most without disclosing this to anyone [6]. By OpenAI’s own estimates, roughly 0.15% of its approximately 800 million weekly users have conversations with explicit indicators of suicidal planning or intent, and 0.07% show possible signs of psychosis or mania, figures that translate to over a million people in acute distress interacting with a general-purpose chatbot each week [7]. This use is often unsupervised, informal, and lacking basic safety measures and an adequate evidence base.

### From Scripted Logic to Dynamic Generation: The Rise of LLM-Powered Systems

The emergence of general-purpose LLMs such as ChatGPT, Claude (Anthropic), and Gemini (Google) has catalyzed a new generation of mental health AI tools by enabling dynamic interactions rather than scripted flows. Starting with the release of GPT-3 in 2020, LLMs ushered in new design possibilities that allowed developers to build more engaging, customizable digital mental health interventions [8]. This shift away from rule-based chatbots toward probabilistic, generative systems accelerated the proliferation of clinical and wellness-oriented tools. Unlike rule-based predecessors, which relied on fixed decision trees triggered by specific user entries, LLM-powered systems generate original, unscripted responses based on user input, context, and system instructions [2,9-11]. Through deep learning architectures trained on large datasets, these systems allowed for greater flexibility and adaptation in real time [2,11].

### New Capabilities, New Expectations

These structural changes have enabled several advances that differentiate LLM-based tools from prior generations. First, they offer highly personalized responses rather than guiding all users through the same conversational path [8,10,12]. Second, they are capable of generating language that appears humanlike [10,11,13], including features such as empathy [10,12], which can enhance users’ sense of natural dialogue [9] and trust in the interlocutor. Third, these qualities may improve engagement, as users are more likely to continue interacting with systems that feel responsive and attuned [8,13]. Finally, LLMs are better equipped to manage complex input [12,14], including emotionally rich user statements and extended back-and-forth exchanges, which often overwhelmed rule-based systems and contributed to user frustration [14]. Together, these capabilities have increased the appeal of LLM-powered tools across mental health contexts and shaped expectations: users increasingly anticipate emotionally intelligent, context-aware support, and developers are responding with tools designed to emulate therapeutic experiences.

### Decline of Rule-Based Systems and the Generative Pivot

Notable examples of rule-based chatbots include Woebot (Woebot Health), Wysa (Touchkin eServices), and Tess (X2AI). Woebot investigated the integration of GenAI elements [8], whereas Wysa, originally rule-based, now combines “Generative AI technology in a deterministic framework” [15]. Additionally, Youper emerged as a hybrid model combining rule-based scaffolding with generative elements [13,16]. Overall, the adoption of LLMs has driven a shift toward generative conversational agents, which now form the foundation of many new tools. The discontinuation of the Woebot app in 2025, seen by many as a pioneer and the first “therapy chatbot,” illustrates this transition rather dramatically. Founded in 2017, Woebot simulated natural dialogue but appears to have remained reliant on scripted logic in its commercial deployment. Its sunset, despite “first-mover advantage” and much fundraising success, reflects both growing regulatory uncertainty and a broader preference for more adaptive, GenAI-enabled systems [17].

The generative pivot also carries risks that early evaluations have begun to document. Moore et al [18] found that widely used LLMs expressed stigma toward certain psychiatric conditions, most strongly in alcohol dependence and schizophrenia, and responded inappropriately to roughly one in 5 clinically sensitive prompts, performing worst on delusions, while licensed therapists responded appropriately 93% of the time to the same stimuli, and newer, larger models showed no consistent improvement. Relatedly, a qualitative comparison of 4 widely used LLMs documented racial bias in AI-mediated psychiatric diagnosis and treatment recommendations [19]. Crisis handling shows the same pattern at higher stakes: in a benchmark of 5 general-purpose models against a clinician-informed crisis taxonomy, harmful responses were rare but nonzero and unevenly distributed across models and crisis categories, and the authors argue that tail risk, rather than average performance, is the relevant safety metric for this domain [20]. Findings of this kind are what an evaluation framework must be built to detect: stigma, bias, and crisis-response performance should be core criteria.

### LLM-Powered Tools Across Consumer and Academic Settings

LLM-based mental health chatbots are now widely accessible through company platforms and specialized marketplaces, offering fine-tuned models adapted for specific use cases using custom instructions, additional data, or optimization methods [18]. Some are presented as general wellness companions, while others adopt therapeutic framing or professional-sounding titles. Character.ai’s “Licensed CBT Therapist,” for example, has reportedly hosted nearly 20 million user chats [18]. Other tools include Ebb (Headspace) [21], Nova (Unmind) [22], Ash (Slingshot AI) [23], Mind (Sword Health) [24], and Sunnie (Flourish Science) [25].

More academic prototypes have focused on the administration of standardized symptom screeners (eg, HopeBot [University College London], which delivers the Patient Health Questionnaire-9 [PHQ-9], including a standard item on suicidal ideation [26]; and SouLLMate [Texas A&M University-Corpus Christi], which delivers the PHQ-8 and includes a separate suicide-risk detection feature [27]); cognitive-behavioral therapy delivery (Psyfy [9]; Cady [Karlsruhe Institute of Technology and University of Greifswald] [28]; CaiTI [Columbia University] [29]); third-wave cognitive-behavioral therapy delivery [30]; and emotionally supportive dialogue (ELY [Rutgers University] [31]; Well-Mind ChatGPT [Rutgers University] [32]).

These implementations raise a measurement question that the field has not resolved. The psychometric properties of instruments such as the PHQ-9 were tested under standardized administration, with fixed item wording, order, and response format. A probabilistic model that paraphrases items, reorders them, or embeds them in surrounding conversation may alter how respondents interpret and answer them, and scores obtained this way cannot be assumed to be equivalent to those from standard administration. Until equivalence studies comparing LLM-administered and standard formats are available, results from conversational screeners should be treated as provisional, and evaluation frameworks should require evidence of measurement equivalence for any tool that administers validated instruments.

Research into the efficacy and safety of these tools remains in its infancy. One randomized controlled trial of a fully GenAI mental health chatbot evaluated Therabot (Dartmouth College) in 210 adults with major depressive disorder, generalized anxiety disorder, or clinically at high-risk for feeding and eating disorders, and assessed them based on self-report measures, including the PHQ-9, the Generalized Anxiety Disorder Questionnaire-IV, and the Stanford-Washington University Eating Disorder Screen [30]. The intervention consisted of daily open-ended, LLM-driven conversations grounded in third-wave cognitive-behavioral therapy techniques. All AI-generated messages underwent clinician review before reaching participants, which limits what the trial can establish about the tool’s performance without human oversight. Compared with a waitlist control, users showed significantly greater symptom reductions at weeks 4 and 8. Participants sent an average of 260 messages over 24 days, and therapeutic alliance, assessed via the Working Alliance Inventory–Short Revised, was comparable to outpatient provider norms. However, several factors limit the study’s conclusions, including its self-report, unblinded nature; a waitlist comparison that cannot separate treatment effects from expectancy and attention; novelty effects that can influence results; the study’s short duration (8 wk); and the lack of independent replication to date. Overall, despite such early efforts at systematic testing, these products remain largely unstudied or understudied, whether originating in consumer or academic settings.

## Frameworks and Evaluation Tools Across Sectors

### Approach and Scope

We adopted a narrative, rather than systematic, review approach to the survey of frameworks informing our position paper. Frameworks and guidelines were identified through an iterative, concept-driven process that combined ongoing monitoring and targeted searches of the peer-reviewed literature (PubMed and Google Scholar), preprints (arXiv and SSRN), publications from professional associations (including the American Psychiatric Association and American Psychological Association), and announcements from regulatory bodies (including the Food and Drug Administration [FDA], World Health Organization [WHO], Medicines and Healthcare products Regulatory Agency [MHRA], Therapeutic Goods Administration [TGA], and Health Canada) and industry, spanning from the release of GPT-4 in March 2023 through August 2026, as well as reference chaining from identified sources. We included evaluative frameworks and formal guidance that specifically address generative or LLM-based tools and target mental health applications. We excluded frameworks limited to rule-based applications and general digital health frameworks lacking mental health-specific criteria, referencing the latter only where they inform the argument. In highlighting specific frameworks, we aimed for a purposive, illustrative sample chosen for their influence and relevance, and to represent differing methods, origins, and intended uses.

### Growing Calls for Frameworks and Standards

GPT-4, OpenAI’s paradigm-shifting LLM, was released in March 2023. Despite the rapid rise in mental health chatbot deployment that followed, ethical evaluation remained limited [33], and several reviews began to call for dedicated frameworks and guidelines, especially around risks related to data privacy, consent, and dependency (overreliance on chatbots in ways that downplay user resilience and discourage agency) [34-36].

### Academic Evaluative Initiatives

Several scholarly initiatives attempted to address this gap by defining safety, ethics, and quality criteria for LLM-based mental health tools. While not exhaustive, the following subset illustrates the breadth of approaches that have emerged across research domains and the resulting patchwork of outcomes. Terminology in this space is inconsistent, so we apply the following distinctions: a framework is an organized set of evaluative domains and criteria; a guideline is directive advice on practice, typically issued by a professional or governmental body; and a checklist is an operational instrument for verifying that specified criteria are met. Several of the approaches reviewed combine these functions.

The Framework for AI Tool Assessment in Mental Health (FAITA-MH), developed by the present authors, was an early, scorable, structured framework for evaluating GenAI mental health tools across 6 domains [37,38]: credibility (clarity and achievability of goals, evidence base, and ability to retain users); user experience (how naturally the tool communicates, how it personalizes interactions, and whether it incorporates feedback); crisis management (whether the tool can detect at-risk situations and provide actionable responses); user agency (whether it provides control over personal data, supports autonomy, and promotes self-efficacy); inclusivity (efforts to mitigate bias); and transparency (disclosure of the tool’s creators, funding, and development). FAITA-MH has limitations: its scoring rubric has not undergone formal psychometric validation; its interrater reliability has not been published; and it does not address implementation within health care systems [39].

Stade et al [39] subsequently introduced the Readiness Evaluation for AI-Mental Health Deployment and Implementation (READI), a structured, nonscorable framework that overlaps with FAITA-MH on criteria such as safety, privacy, engagement, effectiveness, and equity, and it also considers diagnostic and therapy-augmenting tools. However, as a nonscorable readiness framework, it does not support quantitative comparison across tools.

Another structured approach, the MHealth-EVAL framework, uses human evaluators’ interactive simulations of common mental health scenarios to assess mental wellness chatbots on 3 features: appropriateness, trustworthiness, and safety [9]. Its role-played scenario method reported good interrater reliability, although the annotators included the evaluators who generated the conversations, and its published demonstration evaluated a chatbot developed by the framework’s authors.

Focused more narrowly on linguistic quality, the empathy, safety, helpfulness, relevance, and overall quality (ESHRO) framework combines human and automated methodologies to evaluate mental health chatbots across 5 dimensions: empathy, safety, helpfulness, relevance, and overall quality [31]. Its safety check labels responses as unsafe or not, without grading degrees of risk; the authors defer fuller integration of safety into the overall score to future versions, and in their demonstration they evaluated their own chatbot, leaving independent validation an open question.

A panel of experts in AI, psychology, digital therapeutics, and human-computer interaction was behind the Comprehensive Evaluation Scale for LLM-Powered Counseling Chatbots (CES-LCC) [40], which incorporated 9 dimensions: understanding of requests, providing helpful information, clarity and relevance of responses, language quality, trust, emotional support, guidance, memory of previous interactions with users, and overall satisfaction. Its items were derived through a 2-round eDelphi process with 16 experts and have undergone initial internal-consistency validation by the originating team; end users participated in validation rather than in item generation, and independent replication has not been reported.

Unlike the panel-driven CES-LCC, the generative AI for mental health framework [11] emerged from a systematic review of GenAI-powered assessment, therapeutic, and clinician-support tools. The framework encompasses guidelines across 4 core dimensions: data privacy, information integrity, user safety, and ethical governance. As an ethical framework distilled from a systematic review, it specifies what should be evaluated without an accompanying assessment procedure.

Finally, Desage et al [16] and Bunge and Desage [14] introduced a structured evaluation process centered on an updated version of their earlier Thera-Turing Test (TTT), which compares a chatbot’s responses to those of trained therapists. The authors use the TTT as part of a broader process for assessing qualities such as empathy, responsiveness, safety, and cultural awareness across human and AI-generated responses. Its human-benchmark design, in which judges rate conversations blind to whether an AI or a therapist produced them, supports fidelity comparisons but may not surface failure modes without human analogs; its published application to date is by the originating team.

When read side by side (Table 1), the frameworks overlap heavily on safety and appropriateness of responses, diverge on scoring and evaluation methods, and share 3 gaps. First, none reports co-design of its criteria with people with personal experience of mental health challenges. Second, postdeployment surveillance receives limited attention: READI calls for postdeployment evaluation, and the TTT recommends periodic readministration after launch, but none specifies systematic adverse-event surveillance. Third, none includes machinery for its own revision. Where frameworks have been demonstrated or validated at all, this has been done by their originating teams, in 2 cases, on chatbots that the framework authors themselves developed. The fragmentation, in other words, is not primarily disagreement about values; it is duplication at the center and underattention at the edges, which is a problem that a meta-framework is positioned to solve.

**Table 1.** Comparative overview of the reviewed evaluation frameworks and guidance.

Framework	Developers/origin	Type	Domains	Method	Scorable	Personal-experience co-design of criteria	Lifecycle coverage	Validation status
FAITA-MH^a^ [37,38]	Golden and Aboujaoude (academic)	Structured framework	Credibility, user experience, crisis management, user agency, inclusivity, transparency	Criterion-based review	Yes	Not reported	Summative	No formal psychometric validation; no independent replication
READI^b^ [39]	Stade et al (academic)	Readiness/reporting framework	Safety, privacy/confidentiality, equity, effectiveness, engagement, implementation	Structured readiness evaluation and reporting	No	Not reported	Development through postdeployment reporting	Derived from narrative review; no validation study reported
MHealth-EVAL [9]	Chen et al (authors affiliated with Psyfy Inc)	Scenario-based interactive evaluation	Appropriateness, trustworthiness, safety	Evaluators’ role-played scenarios, annotated	Qualitative annotation	Not reported	Summative	Demonstrated by originating team on own chatbot (Psyfy); interrater reliability reported (κ=0.8)
ESHRO^c^ [31]	Sherwani et al (academic)	Multimetric rubric with composite score	Empathy, safety, helpfulness, relevance, overall quality	Human plus automated evaluation; weighted composite (safety scored separately)	Yes	Not reported	Summative	Demonstrated by originating team on own chatbot (ELY)
CES-LCC^d^ [40]	Bolpagni and Gabrielli (academic)	27-item multidimensional scale	Nine dimensions including understanding requests, helpful information, clarity/relevance, language quality, trust, emotional support, guidance, memory, satisfaction	eDelphi-derived scale (16 experts, 2 rounds)	Yes	Not reported (end users in validation only)	Summative	Initial internal-consistency validation by originating team; no independent replication
GenAI4MH^e^ [11]	Wang et al (academic)	Ethical guidance framework	Data privacy and security; information integrity and fairness; user safety; ethical governance and oversight	Distilled from PRISMA^f^ systematic review (79 studies)	No	Not reported	Guidance; no assessment procedure specified	Not applicable (guidance, not an instrument)
TTT^g^ [14,16]	Bunge and Desage (academic)	Comparative blind-judgment test within broader process	Quality of conversations vs therapist benchmark including empathy, responsiveness, safety, cultural awareness	Judges rate conversations blind to AI vs human source	Rating-based	Not reported	Phased: simulated clients through large-sample deployment, with periodic readministration after launch	Illustrative application by originating team; no independent replication

a FAITA-MH: Framework for AI Tool Assessment in Mental Health.

b READI: Readiness Evaluation for AI-Mental Health Deployment and Implementation.

c ESHRO: empathy, safety, helpfulness, relevance, and overall quality.

d CES-LCC: Comprehensive Evaluation Scale for LLM-Powered Counseling Chatbots.

e GenAI4MH: generative AI for mental health.

f PRISMA: Preferred Reporting Items for Systematic Reviews and Meta-Analyses.

g TTT: Thera-Turing Test.

### Professional and Nonprofit Guidance

Professional organizations have begun issuing guidance to navigate the ethical and practical implications of AI use in mental health care. The American Psychological Association produced a tool-evaluation checklist outlining questions to guide the assessment of AI-enabled clinical and administrative tools, including considerations around clinician involvement, clinical evidence, personal data practices, and informed consent [41]. The organization’s June 2025 ethical guidance for AI in the professional practice of health service psychology provides principle-based recommendations for clinicians using or developing AI technologies, including transparency and informed consent, mitigation of bias, data security, accuracy, human oversight, and professional accountability [42]. The American Psychological Association’s Ethics Code itself is undergoing revision, with the update expected to address GenAI [3].

The American Psychiatric Association has also weighed in, publishing a 2024 position statement [43] and a 2025 overview of AI applications in mental health care that outlines standards for the responsible integration of AI in psychiatric care [44]. These documents emphasize evidence-based development, validation, privacy, transparency, user safety, bias mitigation, the inclusion of individuals with personal experience of mental health challenges in tool design, and human oversight.

### Industry Commitments

Increasingly, startups and larger companies developing AI-enabled mental health tools are publicly articulating ethical principles to guide their vision. Headspace delineated a set of AI principles that emphasize proactive safety-risk detection, informed consent, user agency, evidence-based standards, inclusion of clinical experts in product design, and inclusivity across identity dimensions [45]. Other companies have published similar commitments: in late 2025, Lyra Health became the largest company to launch a GenAI product within ongoing therapy, marketing it as “clinical-grade” [46] and publishing its own principles for safe AI use in mental health services [47]. That designation has no agreed definition or external validation standard [48]. Voluntary initiatives of this kind are self-reported and unenforceable, and conflicts of interest complicate reliance on them; they are nonetheless a public benchmark against which conduct can be compared, and their convergence on themes such as safety monitoring, consent, and clinical oversight is itself a signal of emerging norms—and underscores the need for the shared evaluative vocabulary that this article proposes.

### Cross-Sector Collaborations

A growing number of cross-sector initiatives illustrate rising momentum for shared guidance. For example, the Mental Health and AI Field Guide, developed through a collaboration between Grand Challenges Canada, the McKinsey Health Institute, and Google, outlines technical, quality, safety, trust, and governance considerations for implementing AI in mental health programs delivered by nonspecialists [49]. Similarly, while not specifically geared to mental health, Hua et al [50] synthesized insights from prior frameworks and stakeholder input into a structured AI health care chatbot evaluation guide through a collaboration between academic institutions and industry leaders such as Microsoft. Their effort generated 271 evaluation questions across a 3-tiered hierarchy encompassing (1) safety, privacy, and fairness; (2) trustworthiness and usefulness; and (3) design and operational effectiveness.

## Conclusions: Toward a Unified, Evolving Meta-Framework

### Challenges of Coordination and Standardization

While the proliferation of frameworks reflects meaningful progress and a shared commitment to ethically relevant themes, the absence of alignment has, in practice, contributed to confusion through fragmented criteria, inconsistent metrics, and difficulty in comparing tools or prioritizing evaluation strategies. Observing that the current patchwork of approaches is ill-equipped to keep pace with the speed and complexity of development, many researchers have called for standardized, systematic, or benchmarked frameworks, guidelines, or metrics to evaluate GenAI mental health tools [2,9,10,51,52]. Researchers have described an “urgent need” for a unified evaluation approach [2,52] and identified the lack of validated methods as an acute methodological challenge that is limiting much-needed scholarship and formal testing in this area [10,51].

Part of the problem lies in the diverging priorities of the various stakeholders, leading frameworks to be shaped by the incentives of their originating settings: academia may prioritize innovation and impact; health care systems may emphasize privacy, reputation, and cost-effectiveness; and industry may center on scalability, user engagement, and financial upside [39]. Without a coordinated process to reconcile these priorities, evaluation standards remain unevenly applied and difficult to operationalize at scale.

As models have become increasingly sophisticated and tools have expanded beyond chatbots into administrative, diagnostic, and hybrid domains, the limitations of static, domain-bound frameworks have become more apparent. In the words of Bolpagni and Gabrielli [40], “as counseling chatbots become more sophisticated due to the introduction of LLMs, so must the methods used to evaluate them.” This need has become more acute as GenAI capabilities have continued to evolve, complexify, and diversify.

### The Need for Global, Cross-Sector Collaboration

A growing number of researchers and organizations have emphasized the need for multistakeholder collaboration in the development of AI evaluation standards for mental health [2,40,53], calling for greater coordination among developers, researchers, clinicians, ethicists, regulators, and policymakers [54-56]. Stade et al [39] call for a unified evaluation process that spans academic, clinical, and commercial settings to address divergent incentive structures and comparability challenges across sectors. Some recent reviews have also called for standardized frameworks specifically to evaluate mental health AI chatbots [2,9,52], while others have focused on the need for shared metrics and evaluation criteria to assess effectiveness, safety, and clinical potential in research contexts [10]. Still others have emphasized the lack of benchmarked ethical frameworks or accountability mechanisms for clinical implementation, arguing that the absence of such guidance hinders the real-world application of these tools in practice [51,55].

While these calls have significantly advanced the conversation, most remain focused on standardizing evaluation within a single domain, such as chatbot safety, research trial design, or clinical implementation. The emphasis has largely been on fixed criteria, with little attention to how those standards should evolve over time as tools, capabilities, and applications rapidly shift in scope and use. An exception is Chenneville et al [3], who call for regular updates to professional ethical standards for psychologists using GenAI. However, their guidance is focused on licensed clinical use and does not seem to extend to autonomous tools or applications used outside formal oversight. Calls for standardization still lack mechanisms for iteration or broader coordination across modalities, settings, and stakeholder roles.

A consortium of this kind would not start from a blank page, and it should harmonize with transnational instruments already in force. The EU AI Act establishes a risk-based regime under which AI systems that qualify as medical devices are treated as high risk [57]. The WHO has issued guidance on the ethics and governance of AI for health, including guidance specific to large multimodal models (ie, those that also process images and audio alongside text) [58,59]. The UK MHRA is adapting software-as-a-medical-device regulation to AI [60], and Health Canada, the US FDA, and the MHRA have jointly published guiding principles for good machine learning practice [61,62]. Australia’s TGA regulates software-based products, including those using AI, within its medical device framework [63]. The consortium should not duplicate these instruments; rather, it should map its criteria to them, adopt their definitions where possible, and concentrate on what they do not cover, including mental health-specific risks such as therapeutic misconception (the mistaken belief that a supportive tool is providing treatment), crisis handling, and stigma. Because the same product can qualify as a wellness tool in one jurisdiction and a medical device in another, the consortium’s criteria should be indexed to a tool’s function, claims, and user population rather than to any single regime’s categories, with published crosswalks translating those criteria into each jurisdiction’s terms. The preceding instruments are transnational; national implementation guidance is emerging in parallel, as in Canada, where 2 federally established bodies, the Mental Health Commission of Canada and the Canadian Centre on Substance Use and Addiction, have commissioned the country’s first national guidance on AI in mental health and substance use health care [64].

Regulatory fragmentation is already a frustrating reality that would be helped by a framework such as the one we propose. Since 2025, the United States has produced a patchwork of divergent approaches [65]: Illinois and Nevada prohibit AI systems from delivering therapy [66,67], with states including Rhode Island and Maine following in 2026 [68,69]; Utah requires disclosure and privacy safeguards for mental health chatbots without banning them [70]; and California and New York condition operation on crisis detection and referral requirements [71,72]. Many of these laws do not reach general-purpose chatbots that are widely used for mental health support without being marketed for it. Regulatory efforts vary across countries as well (eg, Canada’s proposed Safe Social Media Act [73]). A meta-framework cannot substitute for law, but it can supply the shared evaluative vocabulary that makes divergent regimes comparable and interoperable.

Federal regulators in the United States are moving along a separate track from the states: the US FDA Digital Health Advisory Committee met in November 2025 to consider GenAI-enabled digital mental health medical devices, discussing their benefits, risks to health, and risk mitigations, including premarket evidence and postmarket monitoring considerations [74]. At the time of the meeting, the agency had authorized more than a thousand AI-enabled devices, none with a mental health indication; weeks later, it cleared the first software as a medical device with a patient-facing LLM interface, a narrowly bounded insulin-management tool [75], underscoring that a regulatory pathway for GenAI now exists, while mental health remains without an authorized product. That gap and direction of travel are what the structure proposed here anticipates.

As GenAI tools increasingly appear in clinical environments and expand through new interfaces and functions, including triage, documentation, treatment planning, and modes such as voice, video, and ambient sensing, evaluation frameworks must be able to keep pace in real time. Frameworks must also account for uncertainty, including the unknown ways in which risks may emerge not only from anticipated expansions in scope but also from unanticipated behaviors, whether in the system’s outputs or in how users interact with or adapt the tool.

Public-private funding raises the risk of regulatory capture, the gradual bending of an oversight body toward the interests of those it evaluates; the consortium should be designed against this. Precedent exists: the WHO’s Framework of Engagement with Non-State Actors (FENSA) separates private contributions from its standard-setting work [76]. A legal analysis of FENSA’s early implementation observes that it manages engagement risks on a case-by-case basis through Secretariat discretion, without structural limits on private financial influence and that reforms of this kind reduce, but do not eliminate, the risk of undue private influence [77]. A credible structure would go further. Industry contributions should be pooled in a common fund, with no ability to direct money toward particular evaluations, capped as a share of the total budget, and time-limited so that they lapse unless the nonindustry membership votes to renew them (sunset clauses). Administration should sit with an independent secretariat hosted by a neutral body, such as a university consortium or a WHO-hosted partnership. Funders should be barred from evaluating their own or competing products, reinforced by member conflict-of-interest declarations and mandatory recusal. Evaluators should be protected from funding consequences for unfavorable findings, and funders should not hold veto power over publication of results. A global, multistakeholder membership dilutes any single funder’s leverage, although it brings an asymmetry of its own, because industry can fund sustained staff participation while academic and civil society members typically volunteer their time; membership rules and funding caps protect against both.

These efforts should also recognize the transnational character of technologies that know no borders and cannot be effectively governed by city-, state-, or single-country-level regulations alone, unlike many older interventions and technological tools. Promoting the responsible development and regulation of mental health AI will, therefore, also require international coordination. Several authors have highlighted the importance of global collaboration in establishing common ethical standards and regulations [40,78,79].

### Toward a Dynamic Global Framework for Mental Health AI Evaluation

Taken together, calls for standardization, ethical vigilance, and cross-sector and cross-national collaboration point toward the need for a shared, living framework to evaluate GenAI in mental health. We therefore propose the development of an ever-evolving meta-framework; a shared structure that integrates and updates evaluation criteria across settings, systems, and tools. This framework would be maintained by a global consortium of mental health researchers, clinicians, developers, ethicists, people with personal experience of mental health challenges, regulators, and policymakers. Rather than issuing static or siloed guidelines that risk obsolescence and contribute to the fragmentation of the field, this collective effort would coordinate across domains, respond to emerging use cases, and foster alignment across sectors.

None of the frameworks reviewed here, including our own, was co-designed with people with personal experience of mental health challenges. A standard for tools to which people turn in emotionally vulnerable moments should be shaped by those who have lived through them. That these independently developed efforts share this omission suggests a structural issue, which the consortium we propose could address by making co-design a shared requirement instead of leaving it to each group alone. Initiatives that center personal-experience raters, such as the MindBench platform developed with the National Alliance on Mental Illness (NAMI) [80] and a roadmap from the Society of Digital Psychiatry calling for consensus standards in mental health AI [81], signal movement in this direction; what remains missing is a structure that coordinates across such efforts.

The tools discussed here span a wide risk gradient, from general wellness companions to systems performing functions such as suicide-risk detection. These categories differ in regulatory needs, and a single undifferentiated standard would either overburden low-risk products or underprotect users of high-risk ones. A meta-framework should therefore be tiered: a baseline layer applicable to all conversational mental health tools, covering transparency, privacy, crisis referral, and marketing claims, with evidence and monitoring requirements that scale with risk and claimed function, consistent with the risk-based structure of instruments such as the EU AI Act [57]. Tier assignment should follow the claimed and observed functions rather than the marketing category.

Evaluation methodology also differs by model class. Proprietary models accessed through closed APIs are updated continuously, often without notice, so an evaluation conducted on one version may not describe the system with which users interact weeks later. Open-weight or locally deployed models can be version-pinned and independently audited. A meta-framework should treat evaluations as version-specific: certifications tied to a documented model version and date; developer disclosure of update practices; reevaluation triggered by model updates or observed drift; and, for closed models, continuous postdeployment monitoring in place of a one-time assessment.

Evaluation is also not a single event. Formative evaluation occurs during development; summative evaluation at the point of release or approval; and postdeployment surveillance monitors adverse events and drift once tools are in use. Most frameworks reviewed here concentrate on summative assessment. The meta-framework should govern the criteria for all 3 phases and include a mechanism analogous to international pharmacovigilance, in which national centers pool adverse event reports to detect emerging safety signals, as in the WHO’s Program for International Drug Monitoring [82]. Applied here, this means routine adverse event reporting, shared definitions of what counts as a signal, and thresholds at which a tool is rereviewed or withdrawn. Execution would necessarily run through jurisdiction-specific reporting systems; the consortium’s contribution is common criteria, case definitions, and reporting formats that keep adverse event reports comparable across borders. Even the strongest trial evidence to date required clinician supervision of every response; routine adverse event reporting is the scalable substitute.

Although this article concerns mental health, the pattern it addresses is general and can provide a valuable foundation for evaluating general health chatbots. Across disciplines, GenAI offers practitioners and researchers practical benefits, from easing the documentation burden to accelerating literature synthesis, while direct-to-user applications, whether purpose-built tools or general-purpose systems serving as de facto sources of specialized support when dedicated help is out of reach, pair expanded access with the safety risks of the kind that this paper describes.

Such machinery would not be without precedent: this vision aligns with guidance [3] that the American Psychological Association’s ethics code for professional standards must be able to evolve quickly in response to new technologies.

A living framework needs specified machinery operating at 2 levels: continuous benchmarking of the underlying models, whether general-purpose or purpose-trained, and registry-tracked evaluation of the products built on them. Products on general-purpose models can rely on previously published benchmarks for those models; proprietary models have no public record, so the framework itself must require independent model-level evaluation. Criteria should be version-controlled and published with changelogs, so that a tool evaluated under version 2.1 is distinguishable from one evaluated under version 3.0. Review should follow a fixed cycle, set as frequently as the pace of model change warrants and as stakeholder capacity allows, with off-cycle updates triggered by defined events: a major model release, a documented safety incident, a relevant regulatory change, or accumulated safety signals. Criteria whose evidentiary or technical basis has lapsed should be retired through a documented deprecation process. A public registry recording tool, model, and framework versions, along with evaluation dates, would make assessments interpretable and make their expiration visible. Living benchmarking platforms and consensus guidance efforts already operate elements of this machinery [80].

Governance requires the same specificity: defined membership terms and selection procedures, published decision rules such as supermajority voting for criteria changes, dispute resolution by escalation to a vote of the full membership, conflict-of-interest declarations with recusal, and republication of each version on a review cycle under a citable identifier such as a version-specific DOI.

As AI continues to reshape care delivery, research, and users’ lives, the goal is to cultivate a process that is interdisciplinary, global, and continuously refined. It must be capable of evolving with the tools it is meant to guide. While questions remain around implementation, governance, and regulatory alignment, establishing a coordinated foundation is a fundamental first step. Concretely, a near-term pathway could proceed as follows: first, an interim multistakeholder working group convened under an existing body with international reach; second, publication of a version 1.0 crosswalk mapping current frameworks’ domains onto a shared taxonomy, making overlaps and gaps explicit; third, a pilot registry of evaluated tools and versions; and fourth, adoption of a fixed review cycle with published governance rules. None of these steps requires new regulation to begin.

## Abbreviations

CES-LCC: Comprehensive Evaluation Scale for LLM-Powered Counseling Chatbots
FAITA-MH: Framework for AI Tool Assessment in Mental Health
FDA: US Food and Drug Administration
FENSA: Framework of Engagement with Non-State Actors
GenAI: generative AI
LLM: large language model
MHRA: Medicines and Healthcare products Regulatory Agency
NAMI: National Alliance on Mental Illness
PHQ-9: Patient Health Questionnaire-9
READI: Readiness Evaluation for AI-Mental Health Deployment and Implementation
TGA: Therapeutic Goods Administration
TTT: Thera-Turing Test
WHO: World Health Organization

## References

[1] Kolding S, Lundin RM, Hansen L, Østergaard SD (2024). Use of generative artificial intelligence (AI) in psychiatry and mental health care: a systematic review. Acta Neuropsychiatr.

[2] Parks A, Travers E, Perera-Delcourt R, Major M, Economides M, Mullan P (2025). Is this chatbot safe and evidence-based? A call for the critical evaluation of generative AI mental health chatbots. J Particip Med.

[3] Chenneville T, Duncan B, Silva G (2024). More questions than answers: ethical considerations at the intersection of psychology and generative artificial intelligence. Transl Issues Psychol Sci.

[4] Rousmaniere T, Zhang Y, Li X, Shah S (2026). Large language models as mental health resources: patterns of use in the United States. Pract Innov.

[5] Bodner R, Lim K, Siddals S, Goldberg S, Torous J (2026). Barriers to understanding how many people use AI for mental health support: an estimate and narrative review. NPJ Digit Public Health.

[6] McBain RK, Cantor JH, Breslau J (2026). AI chatbot use and disclosure for mental health among US adolescents and young adults. JAMA Pediatr.

[7] O’Dowd A (2025). ChatGPT: More than a million users show signs of mental health distress and mania each week, internal data suggest. BMJ.

[8] Campellone TR, Flom M, Montgomery RM (2025). Safety and user experience of a generative artificial intelligence digital mental health intervention: exploratory randomized controlled trial. J Med Internet Res.

[9] Chen L, Preece DA, Sikka P, Gross JJ, Krause B (2024). A framework for evaluating appropriateness, trustworthiness, and safety in mental wellness AI chatbots. arXiv.

[10] Hua Y, Na H, Li Z (2025). A scoping review of large language models for generative tasks in mental health care. NPJ Digit Med.

[11] Wang X, Zhou Y, Zhou G (2025). The application and ethical implication of generative AI in mental health: systematic review. JMIR Ment Health.

[12] Torous J, Linardon J, Goldberg SB (2025). The evolving field of digital mental health: current evidence and implementation issues for smartphone apps, generative artificial intelligence, and virtual reality. World Psychiatry.

[13] Stade EC, Stirman SW, Ungar LH (2024). Large language models could change the future of behavioral healthcare: a proposal for responsible development and evaluation. NPJ Ment Health Res.

[14] Bunge EL, Desage C (2025). A framework for evaluating mental health artificial intelligence-based conversational agents. J Technol Behav Sci.

[15] Generative AI additional notices. Wysa.

[16] Desage C, Bunge B, Bunge EL (2024). A revised framework for evaluating the quality of mental health artificial intelligence-based chatbots. Procedia Comput Sci.

[17] Aguilar M (2025). Why Woebot, a pioneering therapy chatbot, shut down. STAT.

[18] Moore J, Grabb D, Agnew W (2025). Expressing stigma and inappropriate responses prevents llms from safely replacing mental health providers. ACM Conf Fair Accountab Transpar.

[19] Bouguettaya A, Stuart EM, Aboujaoude E (2025). Racial bias in AI-mediated psychiatric diagnosis and treatment: a qualitative comparison of four large language models. NPJ Digit Med.

[20] Arnaiz-Rodriguez A, Baidal M, Derner E (2026). Between help and harm: an evaluation study of mental health crisis handling by large language models. JMIR Ment Health.

[21] (2025). Meet Ebb. Headspace.

[22] (2025). Nova, your mental health agent. Unmind.

[23] (2025). Slingshot launches Ash, the first AI designed for therapy. Business Wire.

[24] AI care for mental health. Sword Health.

[25] (2025). Sunnie AI. Flourish.

[26] Guo Z, Lai A, Ive J (2025). Development and evaluation of Hopebot: an LLM-based chatbot for structured and interactive PHQ-9 depression screening. Research Square.

[27] Guo Q, Tang J, Sun W, Tang H, Shang Y, Wang W (2024). SouLLMate: an adaptive LLM-driven system for advanced mental health support and assessment, based on a systematic application survey. arXiv.

[28] Kuhlmeier FO, Hanschmann L, Rabe M, Luettke S, Brakemeier EL, Maedche A (2025). Combining artificial users and psychotherapist assessment to evaluate large language model-based mental health chatbots. arXiv.

[29] Nie J, Shao H, Fan Y (2026). LLM-based conversational AI therapist for daily functioning screening and psychotherapeutic intervention via everyday smart devices. ACM Trans Comput Healthcare.

[30] Heinz MV, Mackin DM, Trudeau BM (2025). Randomized trial of a generative AI chatbot for mental health treatment. NEJM AI.

[31] Sherwani SK, Khan Z, Samuel J, Kashyap R, Patel KG (2025). ESHRO: an innovative evaluation framework for AI-driven mental health chatbots. SSRN.

[32] Caceres Najarro LA, Lee Y, Toshnazarov KE, Jang Y, Kim H, Noh Y (2023). WMGPT: towards 24/7 online prime counseling with chatgpt. UbiComp/ISWC ’23.

[33] Coghlan S, Leins K, Sheldrick S, Cheong M, Gooding P, D’Alfonso S (2023). To chat or bot to chat: ethical issues with using chatbots in mental health. Digit Health.

[34] Casu M, Triscari S, Battiato S, Guarnera L, Caponnetto P (2024). AI chatbots for mental health: a scoping review of effectiveness, feasibility, and applications. Appl Sci.

[35] Saeidnia HR, Hashemi Fotami SG, Lund B, Ghiasi N (2024). Ethical considerations in artificial intelligence interventions for mental health and well-being: ensuring responsible implementation and impact. Soc Sci.

[36] Asman O, Torous J, Tal A (2025). Responsible design, integration, and use of generative AI in mental health. JMIR Ment Health.

[37] Golden A, Aboujaoude E (2024). Describing the Framework for AI Tool Assessment in Mental Health and applying it to a generative AI obsessive-compulsive disorder platform: tutorial. JMIR Form Res.

[38] Golden A, Aboujaoude E (2024). The Framework for AI Tool Assessment in Mental Health (FAITA - Mental Health): a scale for evaluating AI-powered mental health tools. World Psychiatry.

[39] Stade EC, Eichstaedt JC, Kim JP, Stirman SW (2025). Readiness Evaluation for AI-Mental Health Deployment and Implementation (READI): a review and proposed framework. Technol Mind Behav.

[40] Bolpagni M, Gabrielli S (2025). Development of a Comprehensive Evaluation Scale for LLM-Powered Counseling Chatbots (CES-LCC) using the eDelphi method. Informatics.

[41] (2024). Companion checklist: evaluation of an AI-enabled clinical or administrative tool. https://www.apaservices.org/practice/business/technology/tech-101/evaluating-artificial-intelligence-tool-checklist.pdf.

[42] (2025). Ethical guidance for AI in the professional practice of health service psychology. https://www.apa.org/topics/artificial-intelligence-machine-learning/ethical-guidance-professional-practice.pdf.

[43] (2024). Position statement on the role of augmented intelligence in clinical practice and research. https://www.psychiatry.org/getattachment/a05f1fa4-2016-422c-bc53-5960c47890bb/Position-Statement-Role-of-AI.pdf.

[44] (2025). Applications of artificial intelligence in mental health care. American Psychiatric Association.

[45] (2025). Our AI principles. Headspace.

[46] Aguilar M (2025). Lyra launches ‘clinical-grade’ chatbot amid growing concern about mental health and AI. STAT.

[47] (2025). Lyra health introduces first clinical-grade AI for mental health. Lyra Health.

[48] Hart R (2025). ‘Clinical-grade AI’: a new buzzy AI word that means absolutely nothing. The Verge.

[49] Google for Health, Google.org, Grand Challenges Canada, and McKinsey Health Institute (2025). Mental health and AI field guide. https://www.tasksharing.ai/api/media/file/Mental%20Health%20and%20AI%20Field%20Guide_vPDF.pdf.

[50] Hua Y, Xia W, Bates D (2025). Standardizing and scaffolding health care AI-chatbot evaluation: systematic review. JMIR AI.

[51] Hua Y, Liu F, Yang K (2025). Large language models in mental health care: a scoping review. Curr Treat Options Psychiatry.

[52] Park JI, Abbasian M, Azimi I (2024). Building trust in mental health chatbots: safety metrics and LLM-based evaluation tools. arXiv.

[53] Jin Y, Liu J, Li P (2025). The applications of large language models in mental health: scoping review. J Med Internet Res.

[54] Badawi A, Laskar MT, Huang JX, Raza S, Dolatabadi E (2025). Position: beyond assistance -- reimagining LLMs as ethical and adaptive co-creators in mental health care. arXiv.

[55] Guo Z, Lai A, Thygesen JH, Farrington J, Keen T, Li K (2024). Large language models for mental health applications: systematic review. JMIR Ment Health.

[56] Sezgin E, McKay I (2024). Behavioral health and generative AI: a perspective on future of therapies and patient care. NPJ Ment Health Res.

[57] (2024). Regulation (EU) 2024/1689 of the European Parliament and of the Council of 13 June 2024 laying down harmonised rules on artificial intelligence and amending Regulations (EC) No 300/2008, (EU) No 167/2013, (EU) No 168/2013, (EU) 2018/858, (EU) 2018/1139 and (EU) 2019/2144 and Directives 2014/90/EU, (EU) 2016/797 and (EU) 2020/1828 (Artificial Intelligence Act). http://data.europa.eu/eli/reg/2024/1689/oj.

[58] (2021). Ethics and governance of artificial intelligence for health: WHO guidance. https://iris.who.int/server/api/core/bitstreams/f780d926-4ae3-42ce-a6d6-e898a5562621/content.

[59] (2025). Ethics and governance of artificial intelligence for health: guidance on large multi-modal models. https://iris.who.int/server/api/core/bitstreams/e9e62c65-6045-481e-bd04-20e206bc5039/content.

[60] Medicines and Healthcare products Regulatory Agency (MHRA) (2021). Software and AI as a medical device change programme. GOV.UK.

[61] (2021). Good machine learning practice for medical device development: guiding principles. https://www.fda.gov/media/153486/download?attachment.

[62] (2023). Predetermined change control plans for machine learning-enabled medical devices: guiding principles. https://www.fda.gov/media/173206/download?attachment.

[63] (2026). Understanding how we regulate software-based medical devices. Therapeutic Goods Administration (TGA).

[64] (2025). Canada gets its first national guidance on AI for mental and substance use health. Mental Health Commission of Canada (MHCC).

[65] Shumate JN, Rozenblit E, Flathers M (2025). Governing AI in mental health: 50-state legislative review. JMIR Ment Health.

[66] (2025). Wellness and Oversight for Psychological Resources Act, Illinois Public Act 104-0054. https://www.ilga.gov/legislation/PublicActs/View/104-0054.

[67] (2025). Assembly bill no. 406. Chapter 283. https://archive.leg.state.nv.us/Session/83rd2025/Bills/AB/AB406_EN.pdf.

[68] (2026). Oversight of Artificial Intelligence Technology in Mental Health Care Act, R.I. Gen. Laws ch. 40.1-5.5 (2026), enacted by 2026-S 2197 Sub A. https://webserver.rilegislature.gov/BillText26/SenateText26/S2197Aaa.pdf.

[69] (2026). An act to regulate the use of artificial intelligence in providing certain mental health services. https://legislature.maine.gov/legis/bills/getPDF.asp?item=3&paper=HP1397&snum=132.

[70] (2025). Artificial intelligence amendments (Utah House Bill 452). https://le.utah.gov/~2025/bills/static/HB0452.html.

[71] (2025). Senate Bill No. 243. https://leginfo.legislature.ca.gov/faces/billNavClient.xhtml?bill_id=202520260SB243.

[72] (2025). Senate Bill S3008C. https://www.nysenate.gov/legislation/bills/2025/S3008.

[73] (2026). Government of Canada introduces legislation to make social media services and AI chatbots safer for children. Government of Canada.

[74] (2025). 24-hour summary of the Digital Health Advisory Committee November 6, 2025. https://www.fda.gov/media/189618/download.

[75] (2025). 510(k) premarket notification. US Food and Drug Administration (FDA).

[76] (2018). Framework of Engagement with Non-State Actors. https://apps.who.int/gb/bd/PDF/Framework_Engagement_non-State_Actors.pdf.

[77] Berman A (2021). Between participation and capture in international rule-making: the WHO Framework of Engagement with Non-State Actors. Eur J Int Law.

[78] Balcombe L (2023). AI chatbots in digital mental health. Informatics.

[79] Olawade DB, Wada OZ, Odetayo A, David-Olawade AC, Asaolu F, Eberhardt J (2024). Enhancing mental health with artificial intelligence: current trends and future prospects. J Med Surg Public Health.

[80] Dwyer B, Flathers M, Sano A (2025). Mindbench.ai: an actionable platform to evaluate the profile and performance of large language models in a mental healthcare context. NPP Digit Psychiatry Neurosci.

[81] Torous J, Ledley KT, Gorban C (2025). Accelerating digital mental health: the Society of Digital Psychiatry’s three-pronged road map for education, digital navigators, and AI. JMIR Ment Health.

[82] The WHO programme for international drug monitoring. World Health Organization.

